# Mediation Analysis Supports a Causal Relationship between Maternal Hyperglycemia and Placental DNA Methylation Variations at the Leptin Gene Locus and Cord Blood Leptin Levels

**DOI:** 10.3390/ijms21010329

**Published:** 2020-01-03

**Authors:** Valérie Gagné-Ouellet, Edith Breton, Kathrine Thibeault, Carol-Ann Fortin, Andres Cardenas, Renée Guérin, Patrice Perron, Marie-France Hivert, Luigi Bouchard

**Affiliations:** 1 Department of Biochemistry, Université de Sherbrooke, Sherbrooke, QC J1K 2R1, Canada; valerie.gagne.ouellet@usherbrooke.ca (V.G.-O.); Edith.Breton@usherbrooke.ca (E.B.); Kathrine.Thibeault@usherbrooke.ca (K.T.); Carol-Ann.Fortin@usherbrooke.ca (C.-A.F.); Renee.Guerin2@USherbrooke.ca (R.G.); 2 Division of Environmental Health Sciences, School of Public Health, University of California, Berkeley, CA 94720, USA; andres.cardenas@berkeley.edu; 3 Department of Medical Biology, CIUSSS Saguenay-Lac-Saint-Jean—Hôpital Universitaire de Chicoutimi, Saguenay, QC G7H 5H6, Canada; 4 Department of Medicine, Université de Sherbrooke, Sherbrooke, QC J1K 2R1, Canada; patrice.perron@usherbrooke.ca (P.P.); MHIVERT@PARTNERS.ORG (M.-F.H.); 5 Centre de Recherche du CHUS, Sherbrooke, QC J1H 5N4, Canada; 6 Department of Population Medicine, Harvard Pilgrim Health Care Institute, Harvard Medical School, Boston, MA 02215, USA; 7 Diabetes Unit, Massachusetts General Hospital, Boston, MA 02114, USA

**Keywords:** childhood obesity, skinfolds thickness, BMI, epigenetics, fetal programming, maternal hyperglycemia, pregnancy

## Abstract

Changes in fetal DNA methylation (DNAm) of the leptin (*LEP*) gene have been associated with exposure to maternal hyperglycemia, but their links with childhood obesity risk are still unclear. We investigated the association between maternal hyperglycemia, placental *LEP* DNAm (25 5′-C-phosphate-G-3′ (CpG) sites), neonatal leptinemia, and adiposity (i.e., BMI and skinfold thickness (ST) (subscapular (SS) + triceps (TR) skinfold measures, and the ratio of SS:TR) at 3-years-old, in 259 mother–child dyads, from Gen3G birth cohort. We conducted multivariate linear analyses adjusted for gestational age at birth, sex of the child, age at follow-up, and cellular heterogeneity. We assessed the causal role of DNAm in the association between maternal glycemia and childhood outcomes, using mediation analysis. We found three CpGs associated with neonatal leptinemia (*p* ≤ 0.002). Of these, cg05136031 and cg15758240 were also associated with BMI (β = −2.69, *p* = 0.05) and fat distribution (β = −0.581, *p* = 0.05) at 3-years-old, respectively. Maternal glycemia was associated with DNAm at cg15758240 (β = −0.01, *p* = 0.04) and neonatal leptinemia (β = 0.19, *p* = 0.004). DNAm levels at cg15758240 mediates 0.8% of the association between maternal glycemia and neonatal leptinemia (*p* < 0.001). Our results support that DNAm regulation of the leptin pathway in response to maternal glycemia might be involved in programming adiposity in childhood.

## 1. Introduction

Childhood obesity is a growing public health problem [1]. It has been linked to various comorbidities, including type 2 diabetes, asthma, dyslipidemia, and psychosocial disorders, which have been increasing recently [2]. In addition, offspring with obesity are likely to remain obese in adulthood, increasing the risk for the perpetuation of a vicious cycle, leading to obesity [3,4]. Thus, it is critical to identify the mechanisms involved in the pathogenesis of childhood obesity, and more specifically in the context of studies on the Developmental Origin of Health and Disease (DOHaD) hypothesis.

The DOHaD hypothesis postulates that exposure to adverse maternal environment during early development is associated with later onset of numerous chronic disorders over the life course, including obesity [5,6,7,8,9]. Plasticity of the epigenome combined with high rate of cellular divisions and differentiations, and also strong DNA methyltransferases (DNMTs) activity during embryonic and fetal development may be part of the molecular mechanisms implicated in DOHaD [10,11]. DNA methylation (DNAm), the most studied epigenetic modification, has been identified among the potential underlying mechanisms involved in the regulation of many metabolic functions, including leptin (*LEP*) gene expression regulation in the placenta [12,13,14]. Indeed, the placenta has been suggested as a relevant tissue in the field, as it plays a crucial role in materno–fetal exchanges optimizing fetal growth, influences maternal physiology by secreting hormones (e.g., leptin), and has on average lower DNAm levels, suggesting that many genes are actively transcribed to support its metabolic activity [15,16,17].

Leptin, a hormone mainly secreted by white adipocytes, but also by the placenta, is a key player in the regulation of energy expenditure [18]. Leptin inhibits the hypothalamic stimuli for food intake and therefore is highly suspected to be a risk factor for obesity and insulin resistance when deregulated [19,20]. We have shown before that maternal hyperglycemia is associated—and may be causally implicated—with changes in neonatal offspring DNAm levels at the leptin gene (*LEP*) [21]. However, whether these variations in DNAm at the *LEP* gene locus are linked to future risk of obesity is still unknown. Therefore, we hypothesized that placental DNAm variations at the *LEP* gene locus contribute to the programming of abnormal fat accretion in childhood in response to maternal glucose metabolism changes in pregnancy.

## 2. Results

### 2.1. Sample Characteristics

Characteristics (mean ± SD) of the 262 mothers–child dyads are shown in Table 1. Briefly, on average, mothers’ age was 28.2 ± 4.3-years-old and had an early pregnancy Body mass index (BMI) of 25.4 ± 5.7 kg/m^2^. Gestational age at birth was 39.6 ± 1.0 weeks, with a birthweight of 3.4 ± 0.4 kg. At follow-up, children were aged 40.5 ± 3.0 months and had a BMI of 16.3 ±1.9 kg/m^2^. The sum (Σ) and ratio (suprascapular (SS): tricipital (TR)) of skinfold thickness (ST) were 17.5 ± 4.1 mm and 0.6 ± 0.2 respectively. We observed positive correlations between childhood BMI z-scores and skinfold measures (ΣST: *r* = 0.49 *p* < 0.001; and SS:TR: *r* = 0.11, *p* = 0.03) at 3-years-old. Birthweight was associated with cord blood leptinemia (*r* = 0.33, *p* < 0.001), as well as weight (*r* = 0.33; *p* < 0.001) and BMI z-score (*r* = 0.22; *p* < 0.001) at 3 years old but not with any of the two ST measures (ΣST: *r* = 0.01; *p* = 0.91, SS:TR: *r* = 0.04; *p* = 0.50). Maternal fasting glucose levels measured at the second trimester of pregnancy were associated with cord blood leptinemia (β = 0.19, *p* = 0.004) after adjustment for maternal age and BMI at the first trimester of pregnancy, gestational age at delivery, and sex of the offspring. Alone, second-trimester maternal fasting glucose levels explain 2.7% of the neonatal leptin levels variance (*p* = 0.009). Maternal glycemia during pregnancy was not significantly associated with any of the adiposity markers (i.e., BMI z-scores: *r* = −0.03, *p* = 0.62; ΣST: *r* = 0.04, *p* = 0.53; and SS:TR: *r* = 0.11, *p* = 0.09) measured at 3-years-old

### 2.2. Identification of Potential Functional LEP 5′-C-phosphate-G-3′ (CpG) Sites and Association with Childhood Anthropometric Profile

**Figure 1:**
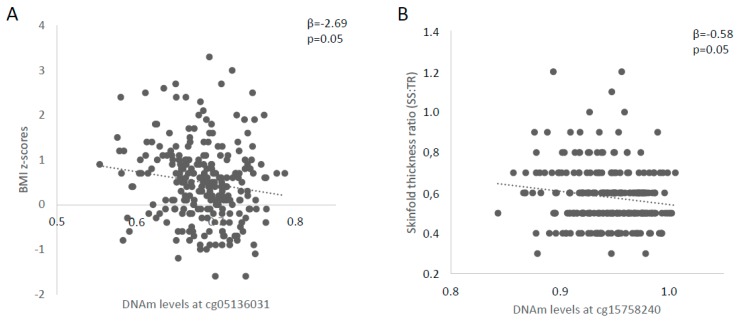
Associations between placental *LEP* DNA methylation (DNAm) and childhood adiposity. Linear regressions were applied to assess the relation between *LEP* DNAm levels at (**A**) cg05136031 and BMI z-scores; and (**B**) cg15758240 and the skinfold thickness ratio (SS:TR), a marker of fat distribution. All *p*-values are adjusted for gestational age at delivery, sex of the offspring, age at follow-up (for skinfold thicknesses) and cell-types composition using ReFACTor. DNAm levels are shown between 0 and 1.

We prioritized CpG sites more likely to have a regulatory role on cord blood leptinemia based on the correlation between DNAm levels and cord blood leptinemia. After Bonferroni correction for multiple testing for nine independent CpG sites or regions (i.e., highly intercorrelated CpGs; significant at *p* < 0.006), methylation levels cg15758240 (*r* = −0.29, *p* < 0.001), cg05136031 (*r* = 0.22, *p* < 0.001), and cg23381058 (*r* = −0.20, *p* = 0.002) were significantly associated with cord blood leptin levels and thus retained for further analyses (Table 2). We then tested associations between DNAm between these three CpG sites and childhood-adiposity-related measures (Table 3). DNAm levels at cg05136031 were associated with BMI z-scores (β = −2.69, *p* = 0.05), whereas those at cg15758240 were associated with SS:TR (β = −0.581, *p* = 0.05) at 3 years of age (Figure 1).

### 2.3. Investigation of Placental DNAm Levels as Mediating Factor between Maternal Glycemia and Adiposity Markers in Offspring

**Figure 2:**
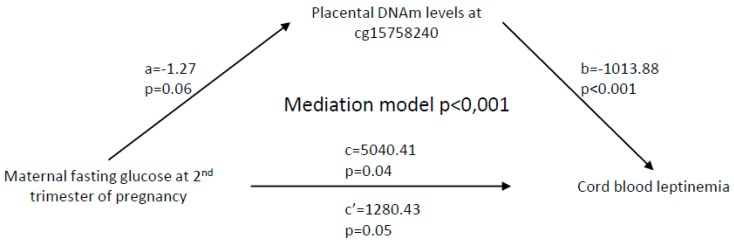
Mediation analysis between maternal fasting glucose at the second trimester of pregnancy, placental *LEP* DNA methylation (DNAm) variations, and neonatal leptin concentrations. Path *a* is the relationship between maternal glycemia and DNAm levels at cg15758240, while path *b* shows the association between DNAm variations and cord blood leptin levels. Path ***c*** is the indirect relationship between maternal glycemia and offspring leptinemia, and *c′* is the direct effect of this relationship when taking into account the placental *LEP* DNAm signature. Gestational age at delivery, sex of the offspring, maternal age and BMI at 1st trimester of pregnancy, and cell-type composition were all included as covariates.

As maternal fasting glucose levels measured at the second trimester of pregnancy were associated with cord blood leptinemia, we therefore tested whether they were also associated with placental DNAm at the *LEP* gene locus and found a significant association with DNAm at cg15758240 (β = −0.01, *p* = 0.04) (Table 4). These associations provided the ground to conduct mediation analysis. The results support a causal relationship between maternal fasting glucose concentrations at second trimester of pregnancy, placental DNAm at cg15758240, and cord blood leptinemia (mediation model *p* < 0.001; Figure 2). Together, DNAm levels at cg15758240 and maternal glycemia explain 13.7% of cord blood leptin levels variance at birth (*p* = 8.5 × 10^−9^), where DNAm alone mediates 0.8% of the effect. We did not test mediation models with BMI-z and skinfold thickness measures as the outcome, since neither the maternal glycemic profile nor the cord blood leptin levels were significantly associated with these adiposity markers in children (data not shown).

## 3. Methods

### 3.1. Participants’ Selection from Gen3G Cohort

We selected participants from the Genetics of Glucose regulation in Gestation and Growth (Gen3G) prospective birth cohort, which we have described previously [22]. Briefly, between January 2010 and June 2013, we recruited women during the first trimester of pregnancy (*n* = 1024), with a follow-up through to delivery (*n* = 862) at the *Centre Hospitalier Universitaire de Sherbrooke* (CHUS). Women less than 18 years old at first visit, with multiple pregnancy, and pre-pregnancy diabetes were excluded. For the current study, we also excluded participants when oral glucose tolerance test (OGTT) data were incomplete, gestational age was <37 weeks (preterm birth), and placental samples as well as body mass index z-scores (BMI-z) and skinfold thickness (ST) measures at 3-years-old were missing. A total of 262 mother–child dyads were retained for these analyses. The Chicoutimi Hospital Ethics Committee approved the project, along with the ethical review board from the *Centre hospitalier universitaire de Sherbrooke*. All women provided a written informed consent before their inclusion in the study, in accordance with the Declaration of Helsinki.

### 3.2. Gen3G Follow-Up During Pregnancy

We collected both data and biological samples during pregnancy and at delivery. Data included medical history, anthropometric measures, and maternal metabolic profile. We used the height and weight to compute maternal body mass index (BMI), using standard formula (kg/m^2^). All mothers performed a 75 g OGTT between gestational weeks 24 and 30, and gestational diabetes mellitus (GDM) was diagnosed according to the Canadian Diabetes Association (CDA) criteria. Only 12 women with GDM were included in the study, as they were treated with a diet only; we excluded women with GDM treated with insulin, to avoid potential confounding impacts of insulin treatment on placenta biology.

### 3.3. Sampling and Measures at Birth

Within 30 min of delivery, well-trained staff collected cord blood and placental tissues, following a standardized procedure. We collected placental biopsies (1 cm^3^) on the fetal side (i.e., 5 cm from the umbilical cord insertion) and kept at −80 °C and in RNALater (Qiagen, USA) until nucleic acids extractions. We measured neonatal leptin levels in cord blood, using ELISA, following manufacturer instructions (B-Bridge International, USA).

### 3.4. Clinical Evaluation of Children

At the 3 years old follow-up, we collected medical history and anthropometric measures in participating children. Briefly, we measured weight on a numeric balance and height, using a stadiometer. We used these measures to compute the child’s BMI, which was used to assess the BMI-z according to the WHO growth-chart reference for boys and girls (WHO Anthro software). We measured ST in the subscapular (SS) and triceps (TR) regions to the nearest 0.1 mm, using a Holtain skinfold caliper (Holtain Ltd., Crosswell, Wales), and computed the sum (ΣST = SS + TR) as a proxy of overall adiposity, as well as the ratio (SS:TR) to reflect fat distribution in 262 participants. A total of 3 participants with extreme BMI (33.6 kg/m^2^) and skinfolds data (ΣST = 8.5 and 48.5 mm) were excluded from the analyses, as they were ˃3 interquartile ranges (IQR) from the median values, and we were not able to confirm the accuracy of the data.

### 3.5. DNA Extraction and Sample Preparation

We extracted DNA and RNA from placental biopsies using the All Prep DNA/RNA/Protein Mini Kit (Qiagen, USA) following the manufacturer’s standard procedure and quantified double-strand DNA using Quant-iT™ PicoGreen™ dsDNA Assay Kit (Qiagen, USA). We preformed sodium-bisulfite conversion of DNA prior to the methylation analyses following recommendations of the EpiTect Bisulfite Kit (Qiagen, USA).

### 3.6. Measurements of DNA Methylation

DNAm levels were quantified in placental tissues by using the Infinium MethylationEPIC BeadChip (Illumina, USA), as described in our previous study [23]. To reduce potential technical bias, we randomly allocated samples in plates and chips. DNAm data from the EPIC Array were implemented in *minfi* library in R, prior to the previously described preprocessing steps [23], which include samples and probes quality controls (QC). We removed samples which failed the QC (*n* = 8), were mismatched on genotype (*n* = 12) or sex (*n* = 1), and were technical duplicates (*n* = 10). We excluded from the analysis probes weakly detected in ≥5% of placenta samples (*n* = 145 probes). We also excluded probes on sex chromosomes (*n* = 19,536), non-CpG probes (*n* = 2839), SNPs-associated probes (*n* = 77,787), probes affected by a single-base extension (*n* = 5435), or SNPs (*n* = 6075) at CpG sites with a minor allele frequency ≥5% as well, as potential cross-hybridization probes (*n* = 34,088) [24].

**Figure 3:**
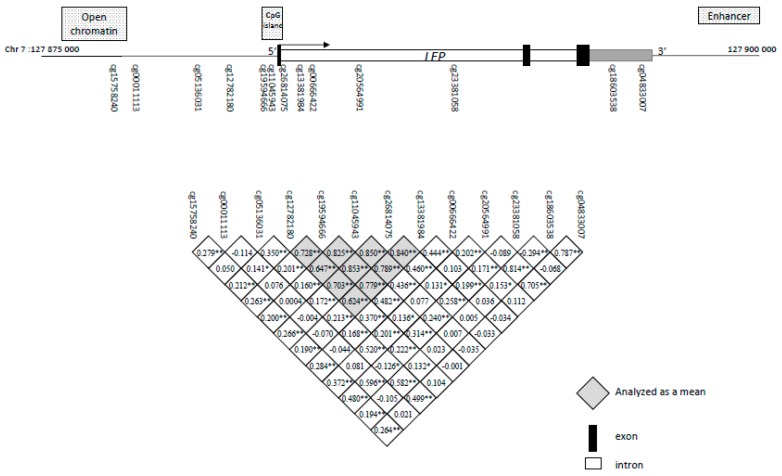
Schematic representation of the *LEP* gene and localization of the epigenotyped CpG sites. The gray square identifies the CpG sites analyzed as a region. * *p* < 0.05; ** *p* > 0.01.

We performed functional normalization that utilizes the control probes, to adjust for technical variability [25]. We used regression on correlated probes (RCP), a method that leverages genomic proximity, to adjust for technical bias from sample plate [26]. After QC, 25 CpG sites upstream or within the *LEP* gene were identified and extracted from the dataset for further analysis, in order to focus on a candidate gene approach. A total of 13 CpG sites (GRCh37/hg19; chr7: 127,876,829–127,894,849), with mean DNAm levels between ≥5% and ≤95%, were included in the analyses, as CpG nearly entirely methylated or unmethylated had been previously shown to have weak interindividual variability [27]. Mean DNAm levels were computed for CpGs showing DNAm correlation (*r* > 0.6) with each other (Figure 3). These highly intercorrelated CpGs (*n* = 5) were analyzed as a region. DNAm outliers were detected based on the IQR and removed from the analysis when they were >3 IQRs from the median values.

### 3.7. Statistical Analyses

The normality of the distributions was tested using the Shapiro–Wilk normality test, and parametric tests were used when log transformation failed to adjust data distribution’s normality. We then investigated the association between offspring’s and mothers’ clinical characteristics (e.g., BMI, SF, and glycemia), using Pearson’s correlation tests. Therefore, we assessed the association between placental *LEP* DNAm and cord blood leptin levels, to identify potentially functional CpG sites. We tested associations by using the Pearson correlation coefficient, and we selected only the statistically significant CpG sites after Bonferroni adjustment for multiple testing (0.05/9 CpGs (i.e., 8 CpGs analyzed individually and 5 CpGs analyzed as a region) = significant at *p* < 0.006) for further analyses (i.e., associations with BMI z-scores and ST measures, using linear regressions). We included gestational age at birth, child’s sex, and age at follow-up (for ST measures) as covariates in multivariate linear regression models. In addition, we conducted an estimation of the placenta cellular heterogeneity by using ReFACTor, which is a reference-free method, included in the models as the first 5 principal components as it was sufficient to bring genomic inflation close to 1 [28]. We did not include maternal smoking during pregnancy, as such a covariate is not associated with either DNAm at the *LEP* gene locus or with childhood adiposity markers. We computed regression coefficients and *p*-values for each CpG, to identify the DNAm sites associated with childhood adiposity, independently of these potential confounding factors. We tested associations between significantly associated adiposity-CpG sites and maternal glycemia during pregnancy, using linear regressions. We adjusted models for maternal age and BMI at the 1st trimester of pregnancy, gestational age at delivery, sex of the child, and age at follow-up and placenta heterogeneity.

### 3.8. Mediation Analysis

We conducted a mediation analysis, using maternal fasting glucose at the 2nd trimester of pregnancy as the exposure, placental *LEP* DNAm levels at cg15758240 as mediators, and cord blood leptinemia as outcome, using the PROCESS procedure created by Hayes, as implemented in the SPSS statistical software (v22.0.0, IBM, USA) [29]. We used 5000 bootstraps to generate estimates, and results were considered significant at *p* ≤ 0.05 [30]. We adjusted the mediation model for gestational age at delivery, sex of the child, maternal age, BMI at 1st trimester of pregnancy, and placenta heterogeneity as covariates. We performed all statistical analyses by using SPSS software.

## 4. Discussion

Accumulating evidence supports that adiposity may be epigenetically regulated and programmed in utero [31,32,33]. However, the biological pathways involved, as well as the influence of the various intrauterine exposures (e.g., maternal obesity and GDM) on epivariations “programming” are yet to be identified. In this study, we provide novel supportive evidence that placental DNAm at the *LEP* gene locus may mediate the association between maternal hyperglycemia and higher cord blood leptin levels, a proxy measure for adiposity at birth, as leptin in cord blood may reflect not only placenta production, but also fetal adipose tissue [18]. To the best of our knowledge, there are only few longitudinal studies, including ours, supporting that epigenetic variations in response to an early exposure to maternal hyperglycemia might include dysregulation of the leptin pathway, potentially leading to a pathophysiological reprogramming of the metabolism and obesity [34].

We previously used a Mendelian randomization approach, using cord blood DNAm levels at the cg12083122 (located in the first intronic region of the *LEP* gene), to investigate the causality link between DNAm variations at the *LEP* gene locus and cord blood leptin levels [21]. In our previous study, we showed that an exposure to maternal hyperglycemia during pregnancy likely modulated neonatal leptinemia, partially through changes in cord blood *LEP* DNAm levels. Interestingly, using placenta and a statistical mediation analysis approach created by Hayes [29], we herein provide additional evidence that a higher maternal glycemia during pregnancy increases cord blood leptin levels, partially through a decrease in leptin DNAm levels in placenta. Our results are consistent with our previous work, using both alternative tissue (i.e., placenta) and CpG site (cg12083122 was excluded from our current analysis, as its mean DNAm levels >95% (97% ± 1%) in placenta tissue) [21].

The cg15758240 provided the more convincing results for a mediating role between maternal glucose levels impacting placental *LEP* DNAm levels, which then contribute to cord blood leptin level regulation and are associated with skinfold thickness at 3-years-old. This CpG is located upstream (−4501 bp) the *LEP* gene (whereas the cg012083122 was located in first intron, +8224 bp), nearby a DNase hypersensitivity cluster (−176 bp) and directly within a region susceptible to histone modifications (H3K27ac mark), an epigenetic modification highly suspected to regulate gene expression through the chromatin remodeling (Supplementary Figure S1) [35]. This CpG site has recently been characterized as an *LEP* interaction region (chr7: 127,854,840–127,881,330). Also based on ENCODE data, cg15758240 is in the vicinity of a number of transcription factor binding sites, including YY1 (−99 bp), CEBPB (−144 bp), USF1 (−164 bp), STAT3 (−184 bp), and GATA2 (−204 bp), which has been previously linked to obesity [36,37,38,39,40], suggesting that cg15758240 is located within a genomic region relevant for both fat accretion and transcriptional regulation activity. Hence, we herein add to the current evidence that the harmful effects of maternal hyperglycemia on offspring adiposity and obesity risk is likely to include epigenetic dysregulation of the leptin gene pathway.

So far, only few studies investigated the epigenetic regulation of the leptin pathway in humans and its impact on childhood adiposity. Although these are mainly cross-sectional studies covering different childhood periods and phenotypes, they showed that whole blood DNAm levels within the leptin gene promoter boundaries were associated with BMI in infancy, along with obesity and insulin resistance in later life [12,13,14,41]. As an example, Tobi et al. reported *LEP* DNAm variations in blood cells collected in 60-year-old men that were exposed to famine periods during in utero development [42]. Interestingly, it was previously shown that *LEP* blood DNAm was lower in obese and insulin-resistant adolescents [12,13,14]. These results, combined with those of Obermann-Borst and colleagues who reported a decrease in the DNAm levels of the *LEP* gene in whole blood cells collected at 17 months in children, in association with increased BMI and birthweight, suggest that the *LEP* DNAm variations could be involved in fetal programming of growth and fat accretion in early childhood [41]. In addition, we and others have previously reported that fetal leptin gene expression and protein levels might be regulated by *LEP* promoter DNAm adaptations to maternal glycemic variations and other metabolic disorders during pregnancy [43,44,45,46]. These studies have better defined the role of epigenetic modifications in the regulation of the leptin gene and highlighted the fetal origins of these epivariations, although none of them were performed in prospective cohorts and they all focused on the *LEP* promoter region.

In our study, placental DNAm levels at the *LEP* gene locus were inversely correlated with both leptin levels at birth (a proxy of neonatal adiposity) and adiposity at 3-years-old, where the strongest association with the latter outcome was found with the skinfold thickness ratio (SS:TR), a marker of fat distribution. More precisely, our results showed that lower placental DNAm levels upstream the *LEP* gene (cg15758240) are associated with the distribution of fat depot in early childhood. Even if this association was modest, these results support that fat distribution might involve early life establishment of epigenetic marks.

In addition, our findings underpin DNAm as a plausible molecular mechanism and the *LEP* gene locus as a determinant of fat accretion and distribution in early childhood, which is in accordance with a previous study supporting how fat distribution (i.e., gynoid vs. android) might be determined before the age of seven [47]. In addition, our association between *LEP* DNAm and maternal hyperglycemia as a marker of childhood fat distribution seemed to be independent of neonatal leptinemia. Interestingly, it was previously reported that neonatal leptin levels might be associated with growth trajectories and childhood BMI [48,49], but other studies showed that both lower and higher leptin levels are associated with weight gain [50,51,52,53,54,55,56,57]. Nevertheless, although the association between neonatal leptinemia and childhood growth and adiposity (e.g., weight gain, BMI, and/or obesity) has been previously reported [48,49,50,51,52,53,54,55,56,57,58], the impacts of altered neonatal leptin levels on fat distribution during childhood still need to be clearly defined.

### Strengths and Limitations

We reported results from a longitudinal study with placenta biopsies combined with a gold-standard technology (i.e., MethylationEPIC array), which provided strong evidence that maternal hyperglycemia contributes to epigenetic dysregulation of neonatal leptinemia, and this is clearly among the strengths of our study. However, we did not show a clear impact on childhood fat accretion and distribution at 3 years of age, maybe due to a lack of power. Consistent with our hypothesis, the assessment of DNAm in adipose tissue at birth and at 3-years-old, for example, would have been clear, but was not feasible for obvious ethical reasons. Still, the investigation of placenta is nonetheless interesting, as this tissue is involved in the secretion of leptin but could also reflect the methylation pattern of other tissues/cell types of common developmental origin [18,59]. Furthermore, the measurement of circulating leptin levels at three years would have made it possible to document the potential temporal stability of the reported DNAm marks and to determine to what extent these fetal origin marks could have an impact later in life.

## 5. Conclusions

In this study, we provide novel evidence supporting the mediating effects of placental *LEP* DNAm dysregulation in response to maternal hyperglycemia during pregnancy on neonatal leptin levels, a proxy for adiposity at birth. Our findings highlight the implication of DNAm as a potential mechanism of fetal programming of adiposity, which might include epigenetic alterations of the leptin gene. Although we also report associations between placental DNAm at the *LEP* gene locus and adiposity at 3-years-old, the causal relationship using mediation analysis could not be tested. Still, these findings underscore the need to look further into the fetal epigenetic programming of childhood obesity.

## Figures and Tables

**Table 1:** Clinical characteristics of the mother–child dyads from the Genetics of Glucose regulation in Gestation and Growth (Gen3G) cohort.

Participant Clinical Data *n* = 262	Mean ± SD
Maternal characteristics	
1st trimester of pregnancy	
Age (years)	28.6 ± 4.2
BMI (kg/m^2^)	25.5 ± 5.5
Smoking during pregnancy	
Yes	7.4%
No	91.8%
Glucose 1 h post 50 g GCT (mmol/l)	5.0 ± 2.2
2nd trimester of pregnancy	
Fasting Glucose (mmol/L)	4.2 ± 0.3
Glucose 2 h post 75 g OGTT (mmol/L)	5.9 ± 1.4
Child characteristics	
At birth	
Gestational age at birth (weeks)	39.6 ± 1.0
Sex	
Boys	55%
Girls	45%
Birthweight (kg)	3.4 ± 0.4
Cord blood leptin levels (ng/mL)	15,028 ± 12,791
At 3-years-old	
Age (months)	40.5 ± 3.0
Weight (kg)	15.2 ± 1.7
BMI (kg/m^2^)	16.1 ± 1.2
BMI z-scores	0.5 ± 0.9
Sum of skinfolds thicknesses (mm)^α^	17.5 ± 3.6
Ratio of skinfold thicknesses (SS:TR)^α^	0.6 ± 0.1

BMI: Body mass index; GCT: glucose challenge test; OGTT; OGTT: Oral glucose tolerance test; SS: Suprascapular; TR: Tricipital. n = 259.

**Table 2:** Correlations between placental Leptin (*LEP)* DNA methylation levels and cord blood leptinemia.

CpG Sites from the MethylationEPIC BeadChip	Cord Blood Leptin Levels
cg15758240	*r* = −0.289 *p* < 0.001
cg00011113	*r* = −0.141 *p* = 0.03
cg05136031	*r* = 0.221 *p* < 0.001
Mean for cg12782180, cg19594666, cg11045943, cg26814075, cg13381984	*r* = −0.125 *p* = 0.05
cg00666422	*r* = −0.129 *p* = 0.04
cg20564991	*r* = −0.061 *p* = 0.34
cg23381058	*r* = −0.198 *p* = 0.002
cg18603538	*r* = 0.006 *p* = 0.93
cg04833007	*r* = −0.045 *p* = 0.48

Cpg: 5′-C-phosphate-G-3′. Results statistically significant after Bonferroni adjustment (*p* < 0.05/9 = 0.006).

**Table 3:** Associations between placental *LEP* DNA methylation levels and markers of childhood adiposity.

CpG Sites	BMI z-Scores	Sum of Skinfold Thickness	Ratio of Skinfold Thickness
cg15758240	β = −0.391 *p* = 0.82	β = 9.076 *p* = 0.21	β = −0.581 *p* = 0.05
cg05136031	β = −2.687 *p* = 0.05	β = −1.441 *p* = 0.80	β = −0.083 *p* = 0.72
cg23341058	β = 0.019 *p* = 0.62	β = 3.407 *p* = 0.35	β = 0.015 *p* = 0.92

The sum of skinfold thickness was computed with SS + TR, whereas the ratio is SS:TR. Significant results when *p* ≤ 0.05. All models were adjusted for gestational age at delivery, sex of the offspring, age at follow-up, and cellular heterogeneity.

**Table 4:** Associations between placental *LEP* DNA methylation levels and maternal glycemia during pregnancy.

CpG Sites	Maternal Glucose 1 h Post 50 g GCT at 1st Trimester of Pregnancy	Maternal Fasting Glucose at 2nd Trimester of Pregnancy	Maternal Glucose 2 h Post 75 g OGTT at 2nd Trimester of Pregnancy
cg15758240	β < 0.001 *p* = 0.74	β = −0.013 *p* = 0.04	β < 0.001 *p* = 0.21
cg05136031	β < 0.001 *p* = 0.72	Β < 0.001 *p* = 0.55	Β < 0.001 *p* = 0.94

Significant results when *p* ≤ 0.05. Models were adjusted for maternal age and BMI at 1st trimester of pregnancy, gestational age at delivery, sex of the child, age at follow-up, and placenta heterogeneity.

## Supplementary Materials

Supplementary materials can be found at https://www.mdpi.com/1422-0067/21/1/329/s1.

## Abbreviations

BMI-z	Body mass index z-scores
DNAm	DNA methylation
CpG	5′-C-phosphate-G-3′
DNMT	DNA methyltransferase
DOHaD	Developmental origins of health and diseases
GCT	Glucose challenge test
GDM	Gestational diabetes mellitus
Gen3G	Genetics of Glucose regulation in Gestation and Growth
IQR	Interquartile range
LEP	Leptin
OGTT	Oral glucose tolerance test
RCP	Regression on correlated probes
RIN	RNA integrity number
SS	Suprascapular
ST	Skinfold Thickness
TR	Tricipital

## References

[1] WHO (2017). Report of the Commission on Ending Childhood Obesity: Implementation Plan: Executive Summary.

[2] Sahoo K., Sahoo B., Choudhury A.K., Sofi N.Y., Kumar R., Bhadoria A.S. (2015). Childhood obesity: Causes and consequences. J. Fam. Med. Prim. Care.

[3] Deshmukh-Taskar P., Nicklas T.A., Morales M., Yang S.J., Zakeri I., Berenson G.S. (2006). Tracking of overweight status from childhood to young adulthood: The Bogalusa Heart Study. Eur. J. Clin. Nutr..

[4] Ma R.C.W., Popkin B.M. (2017). Intergenerational diabetes and obesity—A cycle to break?. PLoS Med..

[5] Barker D.J., Osmond C., Kajantie E., Eriksson J.G. (2009). Growth and chronic disease: Findings in the Helsinki Birth Cohort. Ann. Hum. Biol..

[6] Barker D.J. (2004). The developmental origins of chronic adult disease. Acta Paediatr. Suppl..

[7] Barker D.J. (2004). The developmental origins of adult disease. J. Am. Coll. Nutr..

[8] Barker D.J. (2004). Developmental origins of adult health and disease. J. Epidemiol. Community Health.

[9] Barker D.J. (2005). The developmental origins of insulin resistance. Horm. Res..

[10] Foley D.L., Craig J.M., Morley R., Olsson C.A., Dwyer T., Smith K., Saffery R. (2009). Prospects for epigenetic epidemiology. Am. J. Epidemiol..

[11] Dolinoy D.C., Weidman J.R., Waterland R.A., Jirtle R.L. (2006). Maternal genistein alters coat color and protects Avy mouse offspring from obesity by modifying the fetal epigenome. Environ. Health Perspect..

[12] Garcia-Cardona M.C., Huang F., Garcia-Vivas J.M., Lopez-Camarillo C., Del Rio Navarro B.E., Navarro Olivos E., Hong-Chong E., Bolanos-Jimenez F., Marchat L.A. (2014). DNA methylation of leptin and adiponectin promoters in children is reduced by the combined presence of obesity and insulin resistance. Int. J. Obes..

[13] Xia L., Wang C., Lu Y., Fan C., Ding X., Fu H., Qi K. (2014). Time-specific changes in DNA methyltransferases associated with the leptin promoter during the development of obesity. Nutr. Hosp..

[14] Yang M., Sun J.Z., Sun Y.L., You W., Dai J., Li G.S. (2012). Association between leptin gene promoter methylation and type 2 diabetes mellitus (Article in Chinese). Zhonghua Yi Xue Yi Chuan Xue Za Zhi.

[15] Maccani M.A., Marsit C.J. (2009). Epigenetics in the placenta. Am. J. Reprod. Immunol..

[16] Jansson T., Powell T.L. (2007). Role of the placenta in fetal programming: Underlying mechanisms and potential interventional approaches. Clin. Sci..

[17] Sood R., Zehnder J.L., Druzin M.L., Brown P.O. (2006). Gene expression patterns in human placenta. Proc. Natl. Acad. Sci. USA.

[18] Zhou Y., Rui L. (2013). Leptin signaling and leptin resistance. Front. Med..

[19] Morris D.L., Cho K.W., Rui L. (2010). Critical role of the Src homology 2 (SH2) domain of neuronal SH2B1 in the regulation of body weight and glucose homeostasis in mice. Endocrinology.

[20] Coppari R., Bjorbaek C. (2012). Leptin revisited: Its mechanism of action and potential for treating diabetes. Nat. Rev. Drug Discov..

[21] Allard C., Desgagne V., Patenaude J., Lacroix M., Guillemette L., Battista M.C., Doyon M., Menard J., Ardilouze J.L., Perron P. (2015). Mendelian randomization supports causality between maternal hyperglycemia and epigenetic regulation of leptin gene in newborns. Epigenetics.

[22] Guillemette L., Allard C., Lacroix M., Patenaude J., Battista M.C., Doyon M., Moreau J., Menard J., Bouchard L., Ardilouze J.L. (2016). Genetics of Glucose regulation in Gestation and Growth (Gen3G): A prospective prebirth cohort of mother-child pairs in Sherbrooke, Canada. BMJ Open.

[23] Cardenas A., Gagne-Ouellet V., Allard C., Brisson D., Perron P., Bouchard L., Hivert M.F. (2018). Placental DNA Methylation Adaptation to Maternal Glycemic Response in Pregnancy. Diabetes.

[24] Pidsley R., Zotenko E., Peters T.J., Lawrence M.G., Risbridger G.P., Molloy P., Van Djik S., Muhlhausler B., Stirzaker C., Clark S.J. (2016). Critical evaluation of the Illumina MethylationEPIC BeadChip microarray for whole-genome DNA methylation profiling. Genome Biol..

[25] Fortin J.P., Labbe A., Lemire M., Zanke B.W., Hudson T.J., Fertig E.J., Greenwood C.M., Hansen K.D. (2014). Functional normalization of 450k methylation array data improves replication in large cancer studies. Genome Biol..

[26] Johnson W.E., Li C., Rabinovic A. (2007). Adjusting batch effects in microarray expression data using empirical Bayes methods. Biostatistics.

[27] Logue M.W., Smith A.K., Wolf E.J., Maniates H., Stone A., Schichman S.A., McGlinchey R.E., Milberg W., Miller M.W. (2017). The correlation of methylation levels measured using Illumina 450K and EPIC BeadChips in blood samples. Epigenomics.

[28] Rahmani E., Zaitlen N., Baran Y., Eng C., Hu D., Galanter J., Oh S., Burchard E.G., Eskin E., Zou J. (2016). Sparse PCA corrects for cell type heterogeneity in epigenome-wide association studies. Nat. Methods.

[29] Hayes A.F., Rockwood N.J. (2017). Regression-based statistical mediation and moderation analysis in clinical research: Observations, recommendations, and implementation. Behav. Res. Ther..

[30] Preacher K.J., Hayes A.F. (2008). Asymptotic and resampling strategies for assessing and comparing indirect effects in multiple mediator models. Behav. Res. Methods.

[31] Fernandez-Twinn D.S., Hjort L., Novakovic B., Ozanne S.E., Saffery R. (2019). Intrauterine programming of obesity and type 2 diabetes. Diabetologia.

[32] Goyal D., Limesand S.W., Goyal R. (2019). Epigenetic responses and the developmental origins of health and disease. J. Endocrinol..

[33] Lillycrop K.A., Burdge G.C. (2011). Epigenetic changes in early life and future risk of obesity. Int. J. Obes..

[34] Ling C., Ronn T. (2019). Epigenetics in Human Obesity and Type 2 Diabetes. Cell Metab..

[35] Calo E., Wysocka J. (2013). Modification of enhancer chromatin: What, how, and why?. Mol. Cell.

[36] Verdeguer F., Soustek M.S., Hatting M., Blattler S.M., McDonald D., Barrow J.J., Puigserver P. (2015). Brown Adipose YY1 Deficiency Activates Expression of Secreted Proteins Linked to Energy Expenditure and Prevents Diet-Induced Obesity. Mol. Cell. Biol..

[37] Pan D.Z., Garske K.M., Alvarez M., Bhagat Y.V., Boocock J., Nikkola E., Miao Z., Raulerson C.K., Cantor R.M., Civelek M. (2018). Integration of human adipocyte chromosomal interactions with adipose gene expression prioritizes obesity-related genes from GWAS. Nat. Commun..

[38] Laurila P.P., Soronen J., Kooijman S., Forsstrom S., Boon M.R., Surakka I., Kaiharju E., Coomans C.P., Van Den Berg S.A., Autio A. (2016). USF1 deficiency activates brown adipose tissue and improves cardiometabolic health. Sci. Transl. Med..

[39] Liu Y., Xu D., Yin C., Wang S., Wang M., Xiao Y. (2018). IL-10/STAT3 is reduced in childhood obesity with hypertriglyceridemia and is related to triglyceride level in diet-induced obese rats. BMC Endocr. Disord..

[40] Tong Q., Dalgin G., Xu H., Ting C.N., Leiden J.M., Hotamisligil G.S. (2000). Function of GATA transcription factors in preadipocyte-adipocyte transition. Science.

[41] Obermann-Borst S.A., Eilers P.H., Tobi E.W., de Jong F.H., Slagboom P.E., Heijmans B.T., Steegers-Theunissen R.P. (2013). Duration of breastfeeding and gender are associated with methylation of the LEPTIN gene in very young children. Pediatr. Res..

[42] Tobi E.W., Lumey L.H., Talens R.P., Kremer D., Putter H., Stein A.D., Slagboom P.E., Heijmans B.T. (2009). DNA methylation differences after exposure to prenatal famine are common and timing- and sex-specific. Hum. Mol. Genet..

[43] Lesseur C., Armstrong D.A., Paquette A.G., Li Z., Padbury J.F., Marsit C.J. (2014). Maternal obesity and gestational diabetes are associated with placental leptin DNA methylation. Am. J. Obstet. Gynecol..

[44] Bouchard L., Thibault S., Guay S.P., Santure M., Monpetit A., St-Pierre J., Perron P., Brisson D. (2010). Leptin gene epigenetic adaptation to impaired glucose metabolism during pregnancy. Diabetes Care.

[45] Hogg K., Blair J.D., von Dadelszen P., Robinson W.P. (2013). Hypomethylation of the LEP gene in placenta and elevated maternal leptin concentration in early onset pre-eclampsia. Mol. Cell. Endocrinol..

[46] Lesseur C., Armstrong D.A., Paquette A.G., Koestler D.C., Padbury J.F., Marsit C.J. (2013). Tissue-specific Leptin promoter DNA methylation is associated with maternal and infant perinatal factors. Mol. Cell. Endocrinol..

[47] Chrzanowska M., Suder A., Kruszelnicki P. (2012). Tracking and risk of abdominal obesity in the adolescence period in children aged 7–15. The Cracow Longitudinal Growth Study. Am. J. Hum. Biol..

[48] Buck C.O., Eliot M.N., Kelsey K.T., Chen A., Kalkwarf H., Lanphear B.P., Braun J.M. (2019). Neonatal Adipocytokines and Longitudinal Patterns of Childhood Growth. Obesity.

[49] Yeung E.H., Sundaram R., Xie Y., Lawrence D.A. (2018). Newborn adipokines and early childhood growth. Pediatr. Obes..

[50] Chessler S.D., Fujimoto W.Y., Shofer J.B., Boyko E.J., Weigle D.S. (1998). Increased plasma leptin levels are associated with fat accumulation in Japanese Americans. Diabetes.

[51] Chu N.F., Spiegelman D., Yu J., Rifai N., Hotamisligil G.S., Rimm E.B. (2001). Plasma leptin concentrations and four-year weight gain among US men. Int. J. Obes..

[52] Lissner L., Karlsson C., Lindroos A.K., Sjostrom L., Carlsson B., Carlsson L., Bengtsson C. (1999). Birth weight, adulthood BMI, and subsequent weight gain in relation to leptin levels in Swedish women. Obes. Res..

[53] Ahmed M.L., Ong K.K., Morrell D.J., Cox L., Drayer N., Perry L., Preece M.A., Dunger D.B. (1999). Longitudinal study of leptin concentrations during puberty: Sex differences and relationship to changes in body composition. J. Clin. Endocrinol. Metab..

[54] Byrnes S.E., Baur L.A., Bermingham M., Brock K., Steinbeck K. (1999). Leptin and total cholesterol are predictors of weight gain in pre-pubertal children. Int. J. Obes..

[55] Fleisch A.F., Agarwal N., Roberts M.D., Han J.C., Theim K.R., Vexler A., Troendle J., Yanovski S.Z., Yanovski J.A. (2007). Influence of serum leptin on weight and body fat growth in children at high risk for adult obesity. J. Clin. Endocrinol. Metab..

[56] Johnson M.S., Huang T.T., Figueroa-Colon R., Dwyer J.H., Goran M.I. (2001). Influence of leptin on changes in body fat during growth in African American and white children. Obes. Res..

[57] Savoye M., Dziura J., Castle J., DiPietro L., Tamborlane W.V., Caprio S. (2002). Importance of plasma leptin in predicting future weight gain in obese children: A two-and-a-half-year longitudinal study. Int. J. Obes..

[58] Li L.J., Rifas-Shiman S.L., Aris I.M., Mantzoros C., Hivert M.F., Oken E. (2019). Leptin trajectories from birth to mid-childhood and cardio-metabolic health in early adolescence. Metabolism.

[59] Christensen J.L., Wright D.E., Wagers A.J., Weissman I.L. (2004). Circulation and chemotaxis of fetal hematopoietic stem cells. PLoS Biol..

