# Supplementary figures and images for: Mediation Analysis Supports a Causal Relationship between Maternal Hyperglycemia and Placental DNA Methylation Variations at the Leptin Gene Locus and Cord Blood Leptin Levels
Source: Int J Mol Sci. 2020 Jan 3;21(1):329. doi: 10.3390/ijms21010329 (PMC6982090; doi:10.3390/ijms21010329)

## Slide 1
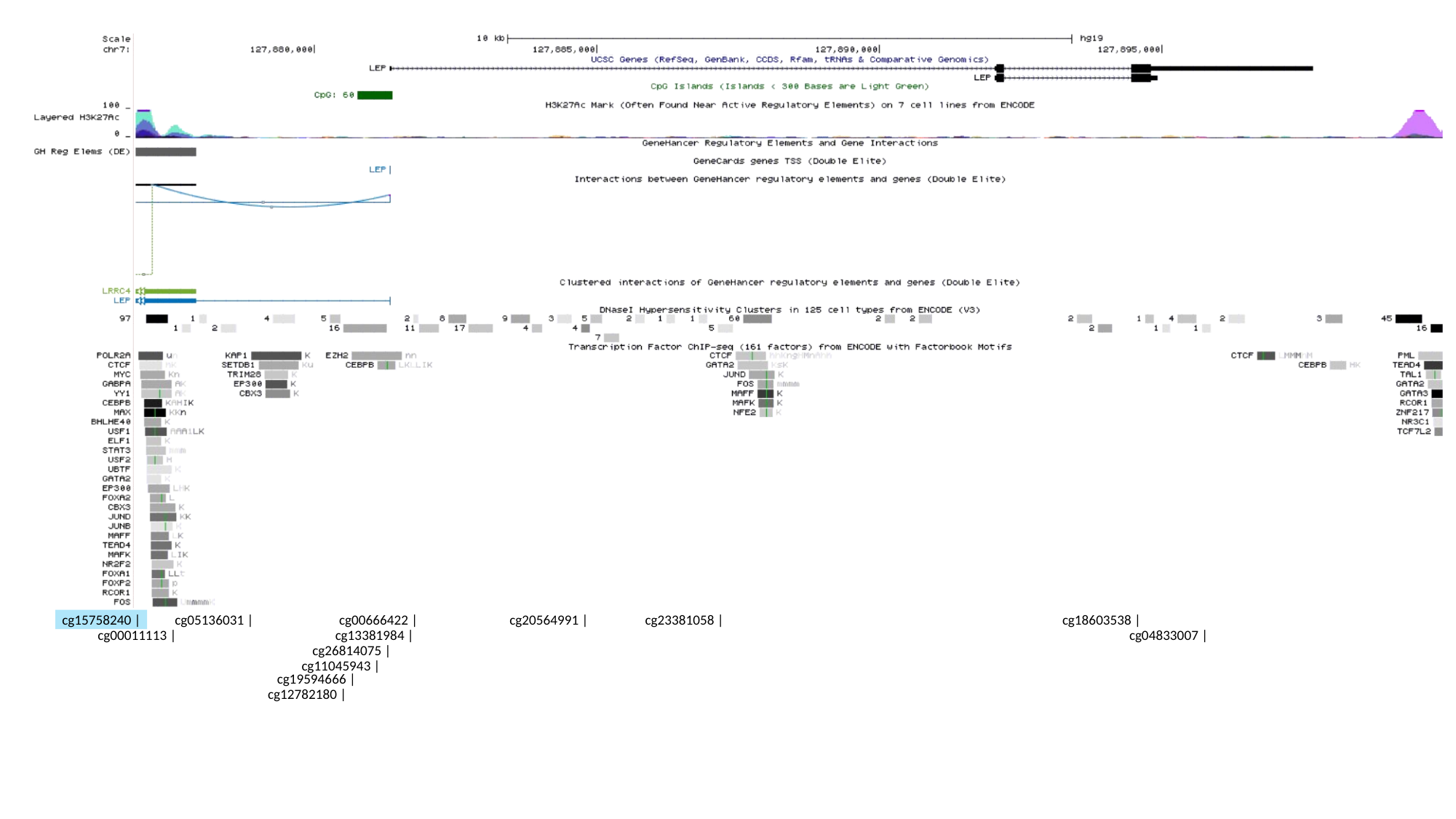

cg15758240 |
cg05136031 |
cg00666422 |
cg20564991 |
cg23381058 |
cg18603538 |
cg00011113 |
cg13381984 |
cg04833007 |
cg26814075 |
cg11045943 |
cg19594666 |
cg12782180 |

Supplement: Supplementary file 1 [file ijms-21-00329-s001.zip › ijms-641697-SI.pptx]
